# Characterization of mutations in hepatitis B virus DNA isolated from Japanese HBsAg-positive blood donors in 2021 and 2022

**DOI:** 10.1007/s00705-024-06016-4

**Published:** 2024-04-18

**Authors:** Ayako Sedohara, Kazuaki Takahashi, Keiko Arai, Kotaro Arizono, Khulan Tuvshinjargal, Makoto Saito, Fumio Nakahara, Takeya Tsutsumi, Kazuhiko Ikeuchi, Eisuke Adachi, Hiroshi Yotsuyanagi

**Affiliations:** 1 Division of Infectious Diseases, Advanced Clinical Research Center, Institute of Medical Science, The University of Tokyo, Tokyo, Japan; 2 Department of Computational Biology and Medical Sciences, Graduate School of Frontier Sciences, The University of Tokyo, Tokyo, Japan; 3 Department of Infectious Disease and Applied Immunology, IMSUT Hospital of The Institute of Medical Science, The University of Tokyo, Tokyo, Japan; 4 Division of Regenerative Medicine, Center for Molecular Medicine, Jichi Medical University, Tochigi, Japan; 5 Department of Infectious Diseases, Faculty of Medicine, The University of Tokyo, Tokyo, Japan

## Abstract

Missense mutations in certain small envelope proteins diminish the efficacy of antibodies. Consequently, tracking the incidence and types of vaccine-escape mutations (VEMs) was crucial both before and after the introduction of universal hepatitis B vaccination in Japan in 2016. In this study, we isolated hepatitis B virus (HBV) DNA from 58 of 169 hepatitis B surface antigen (HBsAg)-positive blood samples from Japanese blood donors and determined the nucleotide sequence encoding the small envelope protein. DNA from six (10%) of the samples had VEMs, but no missense mutations, such as G145R, were detected. Complete HBV genome sequences were obtained from 29 of the 58 samples; the viral genotype was A1 in one (3%), A2 in three (10%), B1 in nine (31%), B2 in five (17%), B4 in one (3%), and C2 in 10 (34%) samples. Tenofovir-resistance mutations were detected in two (7%) samples. In addition, several core promoter mutations, such as 1762A>T and 1764G>A, and a precore nonsense mutation, 1986G>A, which are risk factors for HBV-related chronic liver disease, were detected. These findings provide a baseline for future research and highlight the importance of ongoing monitoring of VEMs and drug resistance mutations in HBV DNA from HBsAg-positive blood donors without HBV antibodies.

**Supplementary Information:**

The online version contains supplementary material available at 10.1007/s00705-024-06016-4.

## Introduction

Hepatitis B virus (HBV) is transmitted via blood and body fluids, vertically by mother-to-child transmission, and horizontally by blood transfusion and sexual transmission. Two billion people are estimated to be infected with HBV worldwide. Most HBV particles are eliminated from the host after infection, leading to a clinical cure. However, approximately 10% (350 million) of infected patients have persistent HBV infection and are positive for hepatitis B surface antigen (HBsAg). Among those with persistent HBV infection, approximately 90% are asymptomatic carriers, whereas 10% develop chronic hepatitis, which can progress to cirrhosis and hepatocellular carcinoma (HCC) [1]. Vaccination is the most effective method of preventing HBV infection. In 1992, the World Health Organization recommended universal hepatitis B vaccination and the inclusion of the hepatitis B vaccine in the Expanded Program on Immunization (EPI).

Among East Asian countries, Japan has a relatively low prevalence of HBV infection; an estimated 1.1–1.4 million people in Japan have persistent HBV infection [2]. In Japan, a project to prevent mother-to-child transmission of HBV was started in 1986. The HBsAg-positivity rate among first-time blood donors aged 16 years in Japan was 0% in 2003 [3]. In addition, the combination of antibody screening methods for hepatitis B core antigen (HBcAg) and nucleic acid amplification testing of individual blood donation has reduced the incidence of transfusion-transmitted HBV infection to 0.19 cases per million [4]. This suggests that most of the unrecognized HBV carriers in Japan who donate blood are likely to have been horizontally infected through sexual transmission.

In Japan, nationwide universal hepatitis B vaccination in infancy only began in 2016. Two hepatitis B vaccines have been approved for use in Japan: Bimmugen, which is derived from genotype C, and Heptavax-II, which is derived from genotype A. These vaccines target the alpha helix, composed of hydrophilic amino acids, of the small envelope protein. However, because this region is mutation-sensitive and genotype-specific, the activity of neutralizing antibodies is reduced when the genotype of the infecting virus strain differs from that of the vaccine strain [5, 6]. The HBsAg-positivity rate did not decrease after the introduction of universal vaccination, suggesting that the current hepatitis B vaccines may prevent the development of hepatitis but not HBV infection [7–9]. This also suggests that more people may become subclinically infected through horizontal transmission and may be unaware of the infection. Although phylogenetic analysis of HBV DNA has been performed using samples from individuals with either acute HBV infection or chronic hepatitis, conducting detailed analyses of HBV DNA from HBsAg-positive blood donors is crucial for tracking the trend in community-acquired infection. In addition, vaccine-escape mutations (VEMs) have been a concern with mass immunization using the hepatitis B vaccine over the years. A case of vertical transmission of HBV infection from an HBV carrier mother with G145R and P120Q mutations was reported in a newborn infant vaccinated with the hepatitis B vaccine immediately after birth [6]. However, there is no evidence of an increase in VEMs since the introduction of universal vaccination; additionally, the association between hepatitis B vaccine administration and an increased incidence of VEMs remains unclear [7]. Most Japanese people do not have antibodies to HBV because universal vaccination in infancy only started in 2016. Phylogenetic and mutational analysis may be used to track the frequency of VEMs before and after the introduction of the universal vaccination program.

Phylogenetic analysis of HBV DNA has shown that HBV can be classified into at least eight genotypes (A–H) based on differences in their sequence. The genotype distribution varies by region. In Japan, the predominant genotypes are A, B, and C, with genotypes C and B accounting for 85% and 12% of HBV carriers, respectively. The incidence of HBV-associated liver damage differs by genotype [9]. In Japan, persistent infection is most commonly associated with genotype A, fulminant hepatitis (FH) is more likely to occur with genotype B, and most patients with HCC are infected with genotype C [10–13]. The 1762A>T/1764G>A double mutation in the core promoter increases HBV replication and causes FH [12, 14–19]. The 1896G>A stop codon mutation in the precore region suppresses the production of e-antigen (HBeAg) and alters the structure of the DNA, resulting in increased HBV replication and HBV-associated liver disease [15, 20–22]. Missense mutations in reverse transcriptase in the polymerase coding region confer resistance to drugs for acute hepatitis [23]. The presence of drug resistance mutations (DRMs) in asymptomatic HBV carriers in Japan may affect the treatment of chronic hepatitis. Therefore, epidemiologically characterizing mutations at the whole-genome sequence level, and not solely focusing on VEM analysis, is crucial.

In Japan, phylogenetic and mutation analysis of HBV has been conducted mainly in patients with acute or chronic hepatitis, and genotyping of blood donors who tested positive for HBsAg but were unaware of their HBV infection has been conducted on only a few sequences of HBV DNA. Therefore, detailed mutation analysis based on whole-genome sequencing is required. In this study, we obtained HBsAg-positive blood samples from the Japanese Red Cross Society, isolated HBV DNA, and performed a detailed analysis of VEMs, DRMs, and core promoter mutations at the whole-genome sequence level. This study revealed the incidence of VEMs, DRMs, and the genotype distribution for HBV in blood samples collected from Japanese donors in 2021 and 2022 and provides a baseline for future follow-up studies.

## Materials and methods

### Source of HBsAg-positive donor blood

We used blood samples from HBsAg-positive blood donors. The Japanese Red Cross Society is the only organization providing a nationwide blood donation service in Japan. Generally, individuals who have not traveled abroad within the past 4 weeks, are in good health, and have not contracted any infectious diseases are eligible to donate blood. Therefore, the target population for the study was HBsAg-positive individuals who were unaware of their HBV infection status. However, we cannot rule out the possibility that some blood donors were aware of their HBV infection status but were unaware that a history of HBV infection made them ineligible to donate blood, even after being cured. Donated blood is screened for specific infections before being used for transfusion. We procured 169 HBsAg-positive blood samples collected in 2021 and 2022 from the Japanese Red Cross Kanto-Koshinetsu Block Blood Center, which receives blood donations from Tokyo, Chiba, and Kanagawa Prefectures. For ethical reasons, medical information, such as age, sex, and HBV treatment history, of the blood donors were not obtained. This study was approved by the Research Ethics Committee of the University of Tokyo (2022-62-1227). The requirement for informed consent was waived because no information on the individual blood donors was included in the analysis.

### HBV DNA extraction from HBsAg-positive blood samples

HBV DNA was extracted from the plasma of HBsAg-positive blood samples using SMITEST EX-R&D (GS-J0201, Medical & Biological Laboratories Co. Ltd., Nagoya, Japan) according to the manufacturer’s instructions. DNA pellets were dissolved in 20 µL of PCR-grade distilled water for quantitative polymerase chain reaction (qPCR), as well as amplification of the full-length and partial sequences of HBV DNA. To amplify partial sequences of HBV DNA encoding the S region, the PCR mix was added directly to the DNA pellet.

### Quantification of HBV DNA using qPCR

**Fig. 1:**
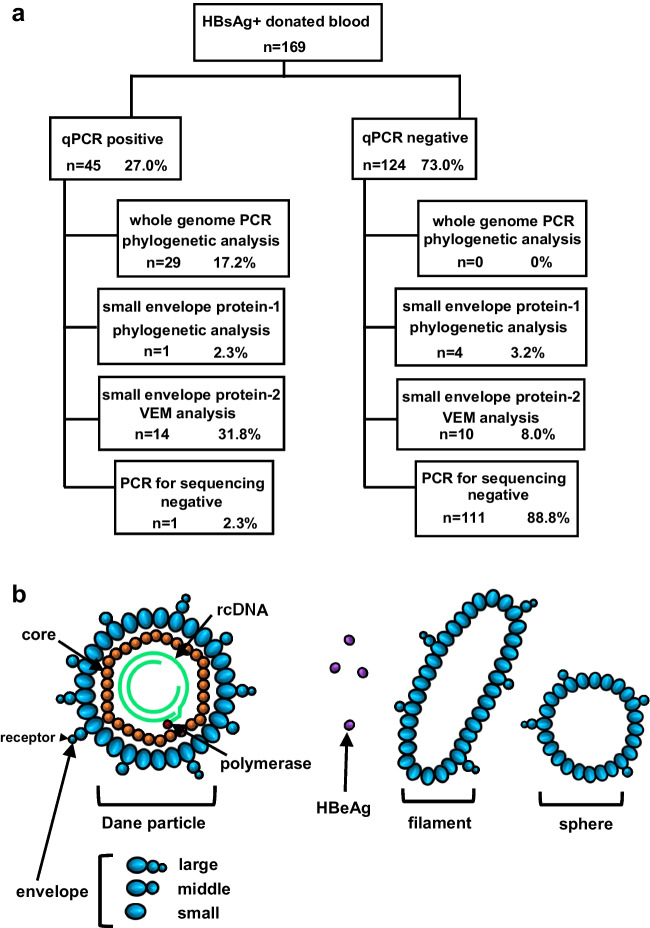
(**a**) Summary of the molecular analysis of the 169 samples from HBsAg-positive blood donors. (**b**) HBV virion. The size of the HBV genome is 3.2 kbp. The HBV genome consists of relaxed circular DNA. HBV genomic DNA is bound to a core protein, which is covered by an envelope protein. The envelope protein is composed of small, middle, and large envelope proteins. The large envelope protein is responsible for binding to hepatocytes. The small envelope protein has hydrophilic amino acids that form the epitope targeted by the hepatitis B vaccine. HBeAg derived from mRNA encoding precore-core protein circulates in the blood. HBeAg is not detected in the presence of a stop codon mutation (1896G>A) in the precore region. Vesicles composed of surface protein called "filaments" or "spheres" are observed in the blood of individuals with HBV infection.

The molecular analysis was performed according to the flowchart shown in Figure 1a. The primer sets and probes used for qPCR are listed in Table 1. THUNDERBIRD Probe qPCR Mix (Toyobo Co., Ltd., Osaka, Japan) was used for qPCR. The qPCR mix was prepared according to the manufacturer’s instructions. Three microliters of DNA solution extracted from plasma was amplified via qPCR using CFX Connect (Bio-Rad, Hercules, CA, USA) by incubating at 95°C for 1 min, followed by 50 cycles of 95°C for 10 s and 55°C for 30 s.

**Table 1:** Primer and probe sequences

Target	Primer name	Sequence	Fw/Rv/probe	1st/2nd PCR
qPCR	HBVTAQ1	GTGTCTGCGGCGTTTTATCA	Fw	1st
	HBVTAQ2	GACAAACGGGCAACATACCTT	Rv	1st
	HBVTAQPR	CCTCTKCATCCTGCTGCTATGCCTCATC	Probe	1st
Full length-1	WA-L	ACTGTTCAAGGGTCCAAGCTGTGC	Fw	1st
	WA-R	AGCAAAAAGTTGCATGGTGCTGGT	Rv	1st
	WA-2L	GGTGGCTTTRGGRCATGGACAT	Fw	2nd
	WA-2R	CAGACCAATTTATGCCTACAGC	Rv	2nd
Full length-2	HBV_F7	ACGTCCTTTGTYTACGTCCCG	Fw	1st
	HBV_R7	GCGAGGCGAGGGAGTTCT	Rv	1st
	HBV_F4	TGCACTTCSCTTCACCTCTGCAC	Fw	2nd
	HBV_R5	GGAGGAGTGCGAATCCACACTCC	Rv	2nd
Small envelope protein-1	P1S	CCTGCTCGTGTTACAGGCGG	Fw	1st
	P2S	TATCGCTGGATGTGTCTGCG	Fw	1st
	P1AS	GCCGRGCAACGGGGTAAAGG	Rv	1st
	P2AS	AARGCAGGATANCCACATTG	Rv	1st
	P3S	CATCCTGCTGCTATGCCTCA	Fw	2nd
	P4S	CAAGGTATGTTGCCCGTTTG	Fw	2nd
	P3AS	GCAAASCCCAAAAGACCCAC	Rv	2nd
	P4AS	CATACTTTCCAATCAATAGG	Rv	2nd
Small envelope protein-2	S1-1	TCGTGTTACAGGCGGGGTTT	Fw	1st
	S1-2	CGAACCACTGAACAAATGGC	Rv	1st
	S2-1	CAAGGTATGTTGCCCGTTTG	Fw	2nd
	S2-2	GGCACTAGTAAACTGAGCCA	Rv	2nd

Fw: forward primer Rv: reverse primer

### Amplification of HBV DNA for sequencing

The primer sets used for amplification of HBV DNA for sequencing are listed in Table 1. KOD One PCR Master Mix (Toyobo) was used for DNA amplification. Three microliters of DNA solution extracted from plasma were used to amplify the target sequence. In cases where the target sequence remained unamplified in 3 µL of the DNA solution, the PCR mix was added directly to the DNA pellet to amplify the target sequence. In the event that the pellet PCR failed to amplify the specific target sequence, the DNA was deemed negative for HBV DNA. Nested PCR was performed for whole-genome amplification. The first PCR comprised the following steps: 95°C for 1 min, followed by 30 cycles of 98°C for 10 s, 60°C for 5 s, and 68°C for 20 s, a final extension at 68°C for 1 min, and hold at 15°C. The second PCR comprised the following steps: 95°C for 1 min, followed by 25 cycles of 98°C for 10 s, 60°C for 5 s, and 68°C for 20 s, a final extension at 68°C for 1 min, and hold at 15°C. Because the structure of the HBV DNA in virions is relaxed circular DNA, sequences of nearly full-length, except for a partial sequence of the X protein coding region, were extracted, followed by extraction of the remaining sequence. A partial sequence encoding the S protein was amplified as follows: The first PCR comprised the following steps: 95°C for 1 min, followed by 30 cycles of 98°C for 10 s, 60°C for 5 s, and 68°C for 10 s, a final extension at 68°C for 1 min, and hold at 15°C. The second PCR comprised the following steps: 95°C for 1 min, followed by 25 cycles of 98°C for 10 s, 60°C for 5 s, and 68°C for 10 s, a final extension at 68°C for 1 min, and hold at 15°C.

### Sequencing, alignment, and phylogenetic analysis

The PCR products were purified using a PCR Purification Kit (QIAGEN, Hilden, Germany) and sequenced using direct (Sanger) sequencing. Sequencing was performed using FASMAC software (Nucleics, Sydney, Australia). The primers used for direct sequencing are listed in Table 2. Each primer (100 µM) was diluted to 2 µM. The results of direct sequencing were checked using ATGC-MAC Ver. 9.0.1 (Genetyx Corp., Tokyo, Japan). The sequences were aligned using Genetyx -MAC Ver. 22.0.1 (Genetyx Corp.). The text files of the alignments were converted to FASTA format using MAFFT Ver. 7 (https://mafft.cbrc.jp/alignment/software/). Phylogenetic analysis was performed by the neighbor-joining (NJ) method using Molecular Evolutionary Genetics Analysis (MEGA) Version 11.0.13 software [24].

**Table 2:** Primers for sequencing of HBV DNA amplified by PCR

Target	Primer name	Sequence
Full length-1	HBV B2466	GTAAAGTTTCCCACCTTATG
	HBV B2830	ATGCYGTAGCTCTTGTTCCC
	P4S	CAAGGTATGTTGCCCGTTTG
	HBV WA-2L	GGTGGCTTTRGGRCATGGACAT
	HBV WA-2R	CAGACCAATTTATGCCTACAGC
	HBV FA3L	CTGCTGGTGGCTCCAGTT
	HBV FA4L	GTATTGGGGGCCAAGTCTGT
	HBV FA2R	GGTATTGTGAGGADDYTTGTCAAC
	HBV 1260	GCCGATCCATACTGCGGAAC
Full length-2	HBV_F6	TGTCAACGACCGACCTTGAG
	HBV_R6	GAGAGTAACTCCACAGWAGCTCC
	HBV_F8	ACTGGGAGGAGYYGGGGGAG
	HBV_R8	CTCCACAGWAGCTCCAAATTCT
Small envelope protein-1	P4AS	CATACTTTCCAATCAATAGG
Small envelope protein-2	S2-1	CAAGGTATGTTGCCCGTTTG
	S2-2	GGCACTAGTAAACTGAGCCA

## Results

### HBV DNA sequences detected in HBsAg-positive blood donor samples

The structure of HBV is shown in Figure 1b. HBV DNA was detected using real-time qPCR in 45 of 169 HBsAg-positive donated blood samples (Fig. 1b, Supplementary Fig. S1). The copy numbers of HBV DNA are listed in Table 3. The whole HBV genome was successfully sequenced from 29 of the 45 samples that tested positive for HBV DNA using qPCR. The pre-surface/surface (preS/S) gene sequences were amplified by PCR from the 16 samples that were negative upon whole-genome sequencing and from 124 samples that were negative for HBV DNA using qPCR, and preS/S sequences were identified in five of these samples. Amplification of the surface region sequence containing VEMs was performed for 135 samples that were negative for preS/S upon PCR testing. The surface region sequence was amplified in 24 samples. Taken together, HBV DNA fragments were detected in 58 (34%) of the 169 HBsAg-positive donated blood samples.

**Table 3:**
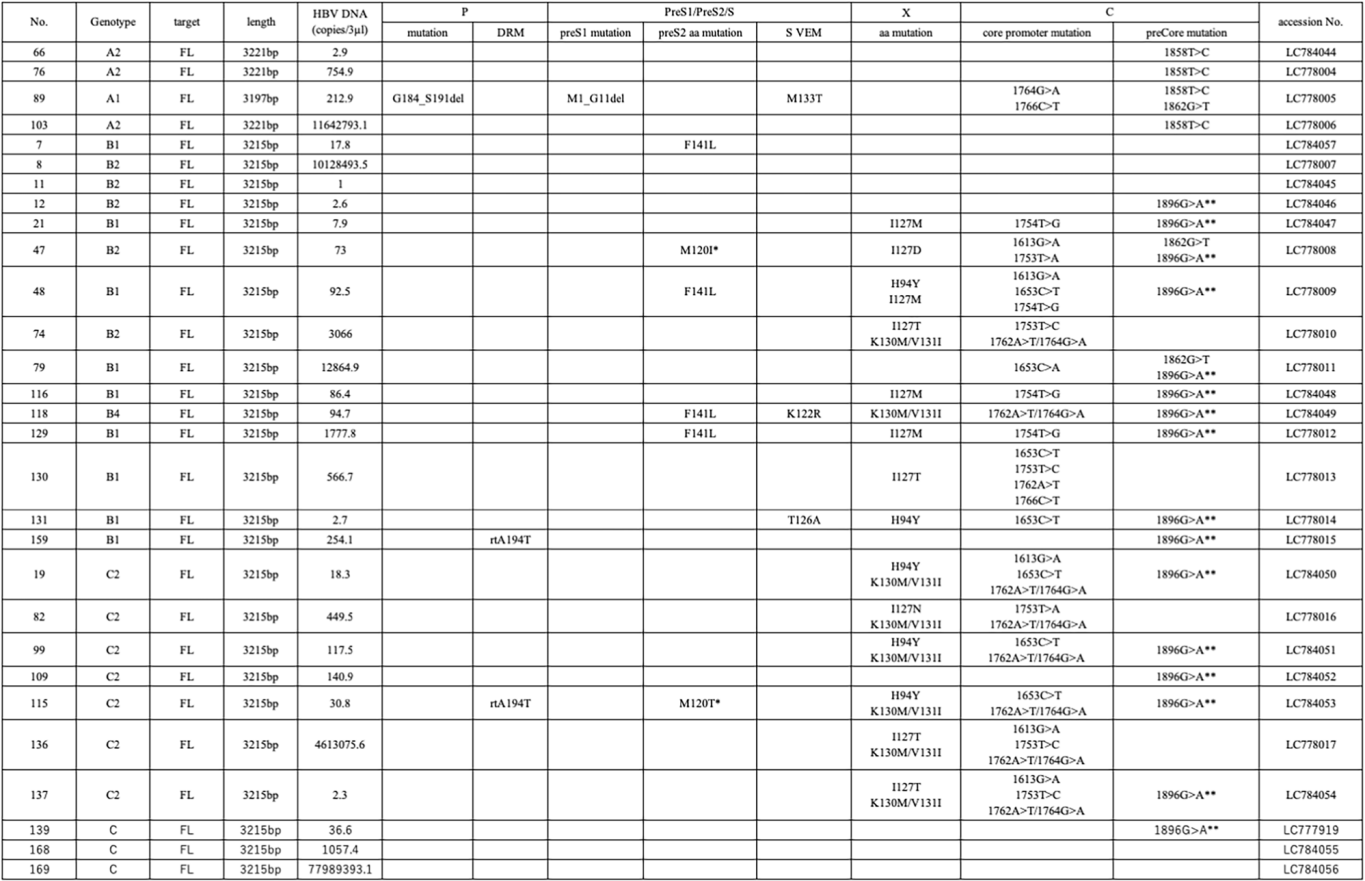
Results of phylogenetic and mutation analysis of whole genome sequences of HBV isolated from HBsAg-positive donated blood

*preS2 start codon mutation **preCore nonsense mutation DRM: drug resistant mutation VEM: vaccine escape mutation

**Fig. 2:**
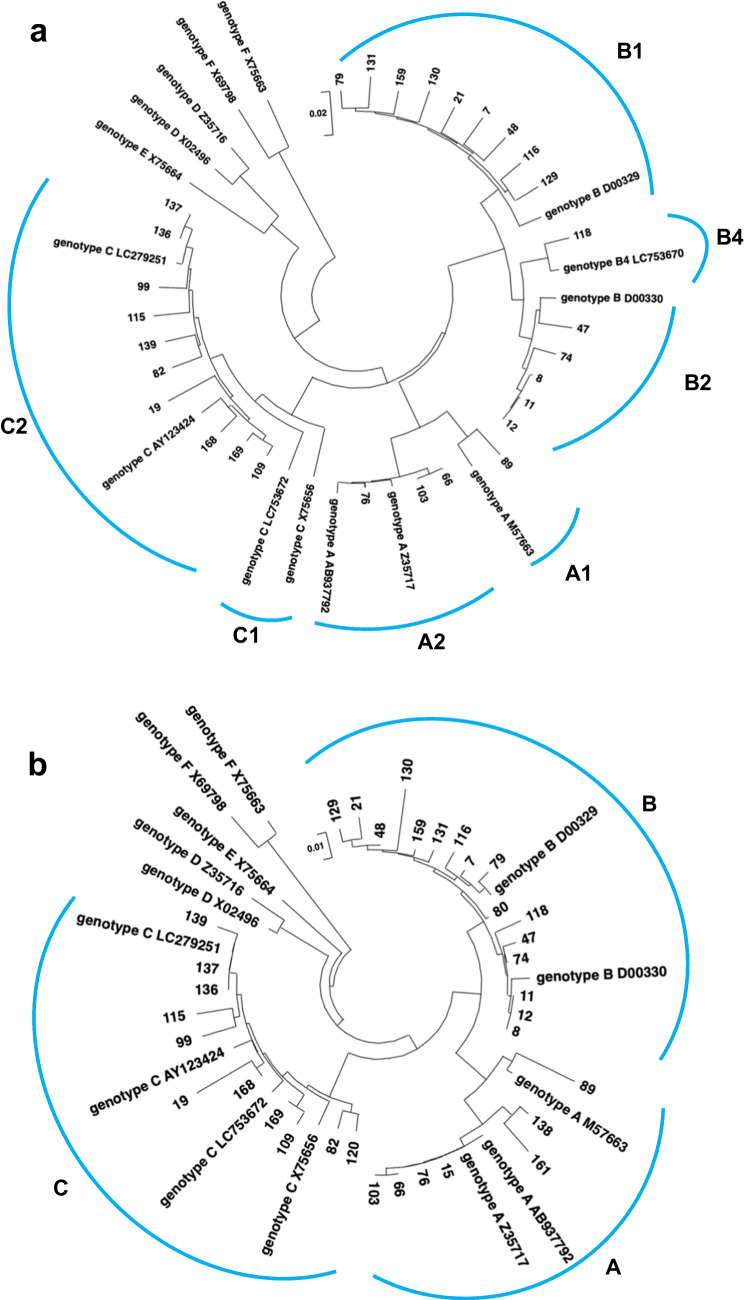
(**a**) Phylogenetic analysis based on HBV whole-genome sequences. (**b**) Phylogenetic analysis of HBV partial sequences encoding the envelope protein

Phylogenetic analysis was performed for the 29 samples with whole-genome sequences (Table 1). The phylogenetic tree is shown in Figure 2a. The genotype distribution in the 29 samples was as follows: 1 (3%) genotype A1 (Asian/African type), 3 (10%) A2 (European type), 9 (31%) B1 (Japanese type), 5 (17%) B2 (Asian type), 1 (3%) B4 (Southeast Asian type), and 10 (34%) C2 (East Asian type). Genotype C1 (Southeast Asia type) was not detected in this cohort. In addition, phylogenetic analysis of the preS/S sequence was performed using the five preS/S PCR-positive samples and 29 samples with whole-genome sequences (Fig. 2b). Phylogenetic analysis of HBV in the 34 samples revealed seven cases of genotype A (21%), 16 of genotype B (47%), and 11 of genotype C (32%).

### Amino acid mutations in the preS/S region in HBV-DNA-positive blood donor samples

**Fig. 3:**
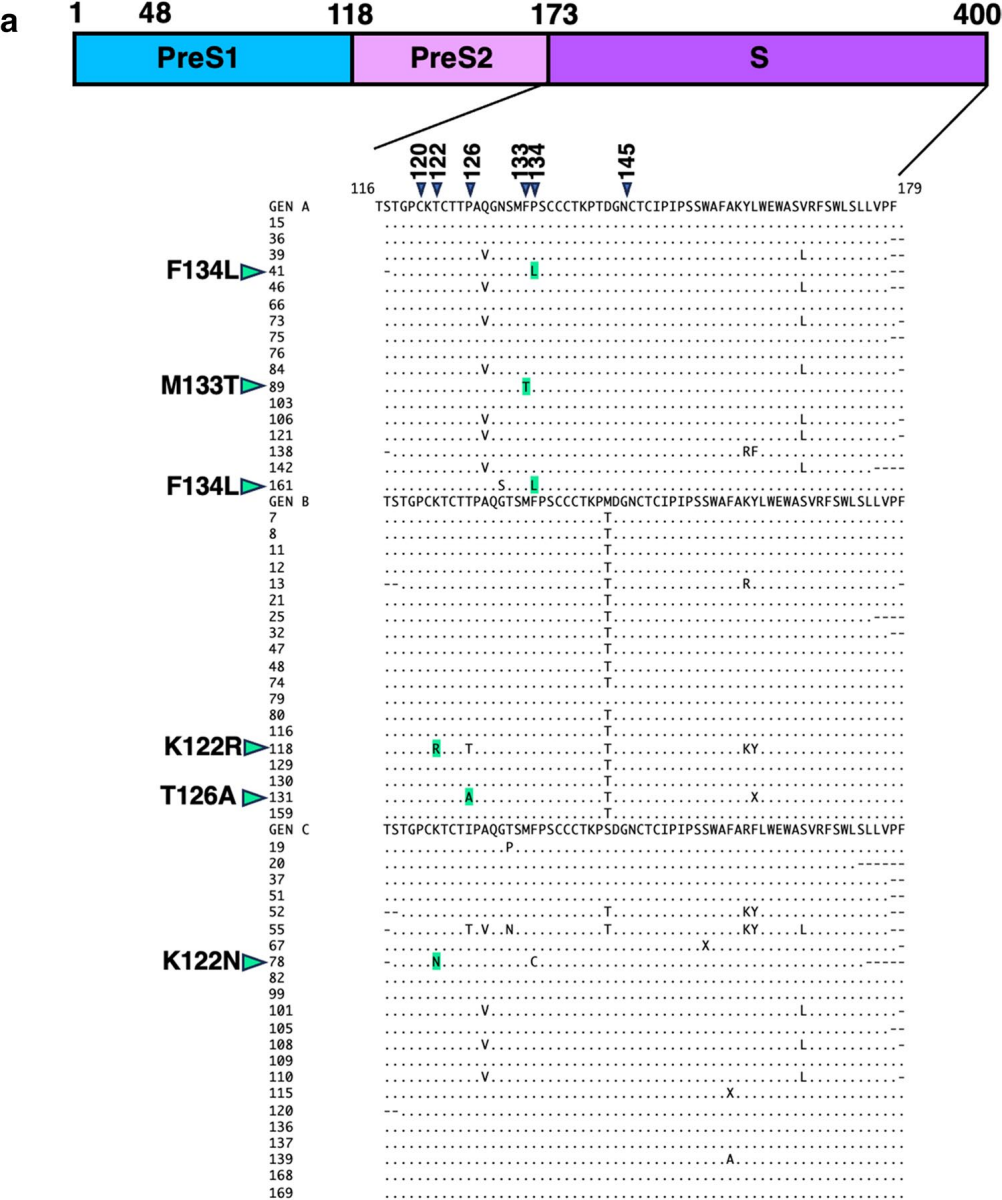

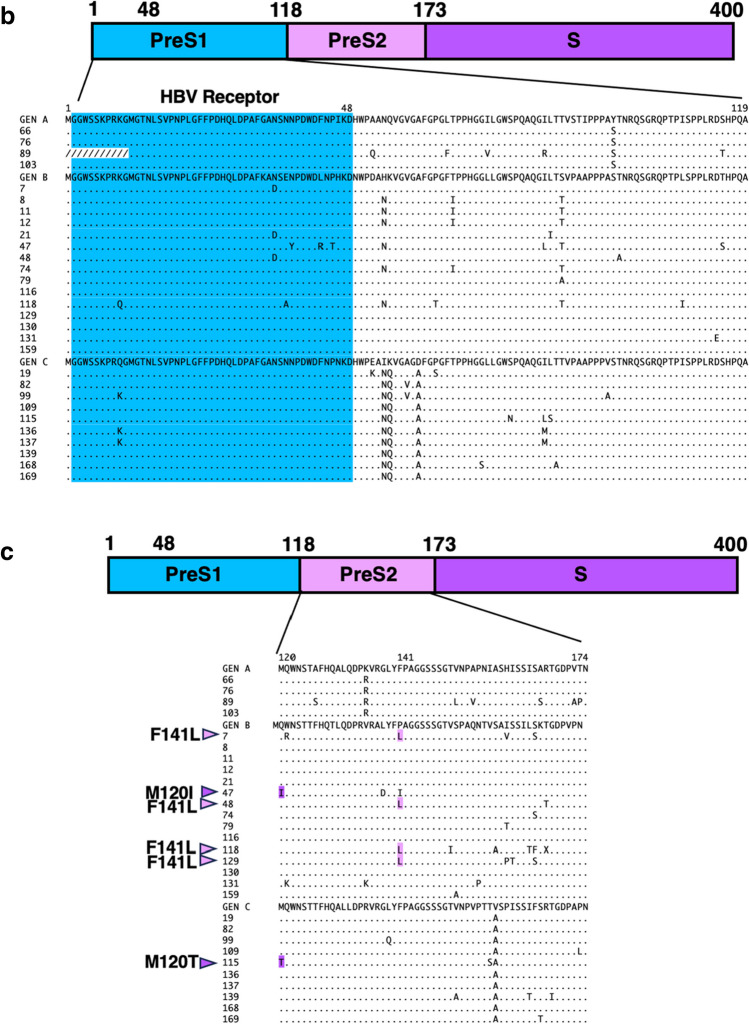
Amino acid mutations observed in the preS1/preS2/S coding region. The diagram shown above the alignment represents the amino acid sequence of the preS1/preS2/S region. The left side of the box represents the N-terminus of the amino acid sequence, and the right side represents the C-terminus. (**a**) Alignment of amino acid sequences of the small envelope protein. Green arrowheads indicate the major vaccine-escape mutations (VEMs). The green symbols indicate amino acid mutations that are known to be VEMs. (**b**) Alignment of amino acid sequences of the preS1 region. The HBV receptor recognition region is composed of amino acids 2–48 at the N-terminus; these amino acids are colored blue. "/" indicates an amino acid deletion. (**c**) Alignment of amino acid sequence of preS2. The purple arrowheads and symbols represent significant amino acid mutations.

Envelope proteins include large, intermediate, and small envelope proteins (Fig. 1b). The schematic diagram in Figure 3a shows the amino acid sequences of the envelope proteins. The small envelope protein is indicated by "S" in the C-terminal domain in the diagram. Small envelope proteins have hydrophilic amino acids that are the target of hepatitis B vaccines [21, 25]. Amino acid sequence alignment allowed several known VEMs to be detected, including K122R, K122N, T126A, M133T, and two F134L mutations, in six of the 58 samples tested (Fig. 3a, Table 3 and 4). Three VEMs (3/17; 18%) were detected in genotype A samples, two (2/19; 10.5%) were detected in genotype B samples, and one VEM (1/22, 4.6%) was detected in genotype C samples. M133T and F134L were found in genotype A, T126A and K122R in genotype B, and K122N in genotype C. No missense mutations commonly present in genotypes were found.

**Table 4:** Results of alignment of HBV small envelope protein amino acid sequences

No.	Genotype	Target	Length	HBV DNA (copies/3µl)	VEM	Accession no.
15	A	Small envelope-1	532 bp	ND		LC777443
36	A	Small envelope-2	188 bp	1.5		LC777892
39	A	Small envelope-2	188 bp	0.4		LC777893
41	A	Small envelope-2	185 bp	0.1	F134L	LC777894
46	A	Small envelope-2	188 bp	2.2		LC777895
73	A	Small envelope-2	189 bp	0.6		LC777896
75	A	Small envelope-2	186 bp	ND		LC777897
84	A	Small envelope-2	191 bp	0.2		LC777898
106	A	Small envelope-2	189 bp	ND		LC777899
121	A	Small envelope-2	189 bp	0.9		LC777900
138	A	Small envelope-1	579 bp	ND		LC777446
142	A	Small envelope-2	180 bp	2.4		LC777901
161	A	Small envelope-1	595 bp	7.9	F134L	LC777447
13	B	Small envelope-2	183 bp	1.2		LC777903
25	B	Small envelope-2	182 bp	ND		LC777905
32	B	Small envelope-2	188 bp	ND		LC777906
80	B	Small envelope-1	515 bp	ND		LC777445
20	C	Small envelope-2	176 bp	0.8		LC777907
37	C	Small envelope-2	188 bp	ND		LC777908
51	C	Small envelope-2	188 bp	1.5		LC777909
52	C	Small envelope-2	182 bp	0.6		LC777910
55	C	Small envelope-2	184 bp	0.9		LC777911
67	C	Small envelope-2	189 bp	1.1		LC777912
78	C	Small envelope-2	174 bp	ND	K122N	LC777913
101	C	Small envelope-2	189 bp	ND		LC777914
105	C	Small envelope-2	188 bp	ND		LC777915
108	C	Small envelope-2	189 bp	ND		LC777916
110	C	Small envelope-2	189 bp	ND		LC777917
120	C	Small envelope-1	512 bp	ND		LC777444

VEM: vaccine escape mutation

The large envelope consists of preS1, preS2, and S proteins (Fig. 3a). The large envelope protein has an HBV receptor encompassing the 48 N-terminal amino acids of preS1 [26]. Alignment of the amino acid sequence of preS1 revealed a deletion mutation in the region encompassing amino acids 1–11, which is important for HBV binding to hepatocytes, in sample number 89 (Fig. 3b). No other amino acid mutations altering the HBV receptor function were observed.

The middle envelope consists of preS2 and S (Fig. 3c). Alignment of the preS2 amino acid sequences revealed two samples with a start codon mutation (M120I) and four samples with a missense mutation (F141L) (Fig. 3c). Both M120I and F141L have been reported to increase the risk of chronic liver diseases, such as cirrhosis and HCC [26, 27].

### Tenofovir-resistance mutations (rtA194T) in HBV DNA-positive blood donor samples

**Fig. 4:**
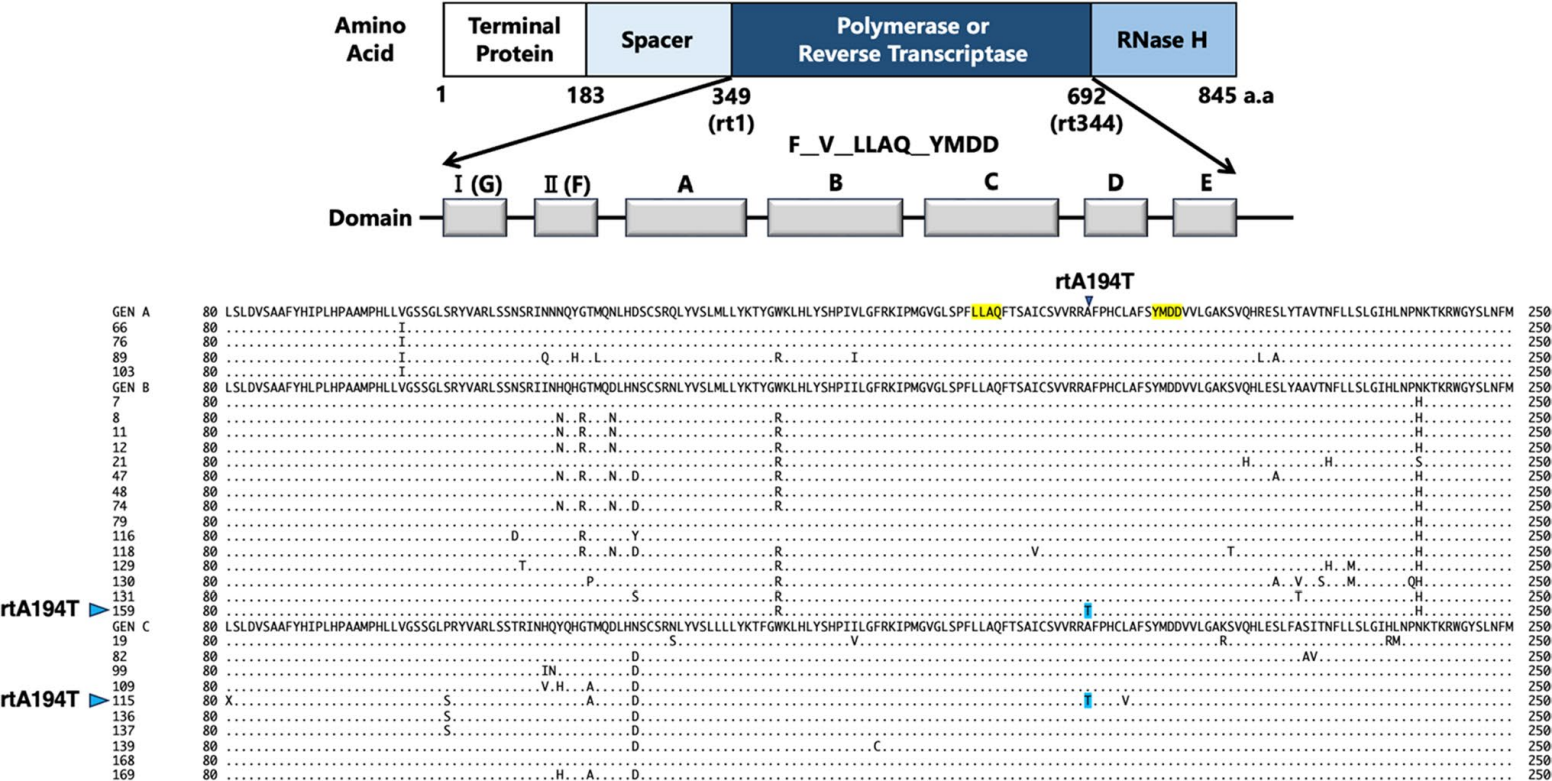
Schematic diagram of the HBV polymerase showing the positions of drug resistance mutations in blood samples from HBsAg-positive donors.. The reverse transcriptase domain comprises amino acids 349–692 (rt1 through rt344) of HBV polymerase. The rtA194T mutation is a tenofovir (TFV)-resistance mutation. The blue symbols indicate TFV-resistance mutations.

When HBV multiplies, the reverse transcriptase encoded in the polymerase region is used to produce pregenomic RNA from HBV DNA. As HBV antiviral drugs target the reverse transcriptase, mutations in this region can lead to drug resistance [28, 29]. Therefore, the 29 samples with whole-genome sequences were tested for DRMs (Fig. 4). HBV polymerase consists of 845 amino acids, with amino acids 349–692 comprising the reverse transcriptase domain. In HBV research, amino acids 349–692 are referred to as reverse transcriptase 1 (rt1) through rt344. Comparison of amino acid sequences revealed HBV DNA with the rtA194T mutation that confers drug resistance to tenofovir in two of the 29 (7%) samples tested.

### Core promoter mutations and precore mutations in the HBV DNA in HBV-DNA-positive blood donor samples

**Fig. 5:**
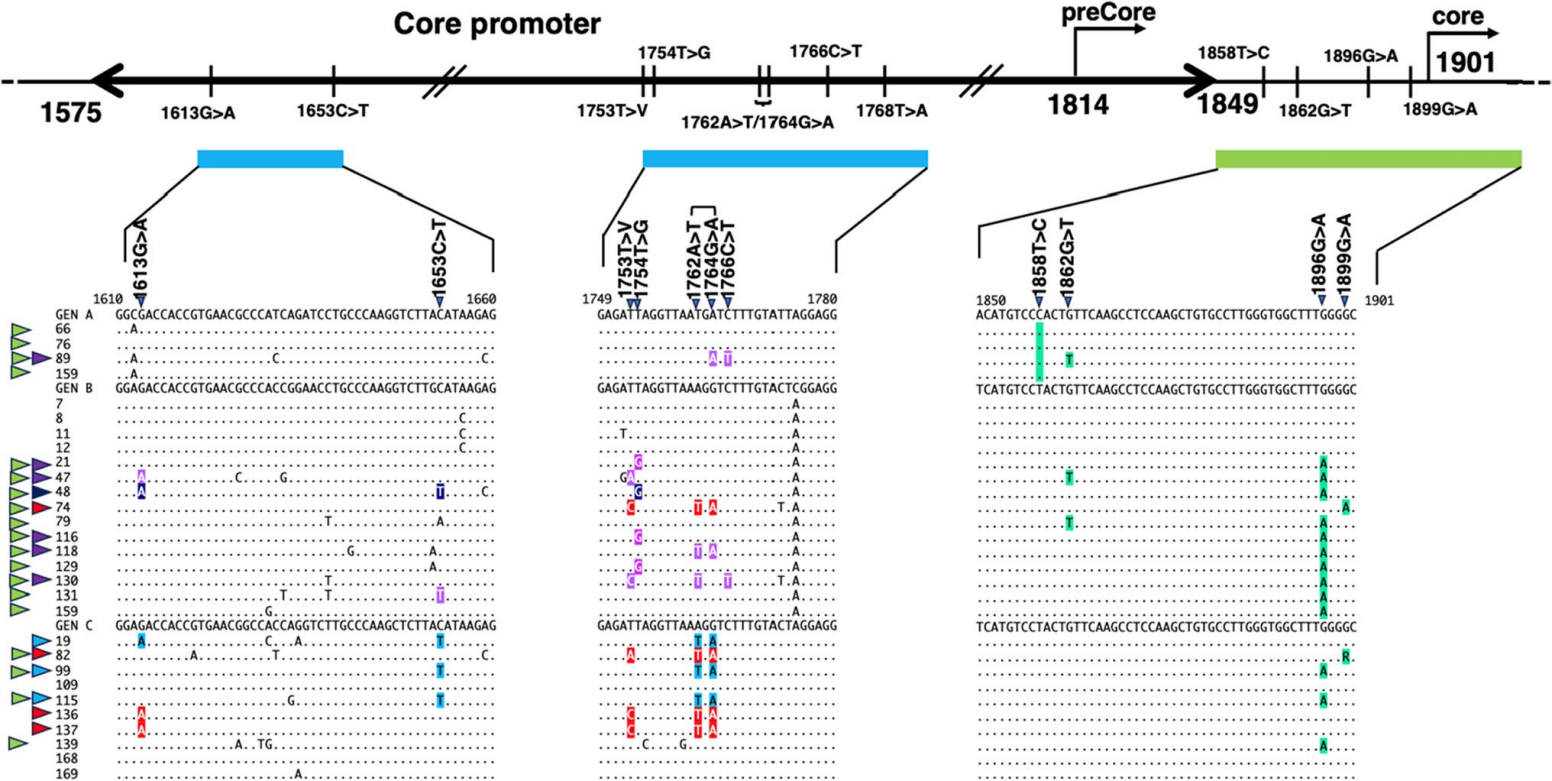
Schematic diagram showing the main nucleic acid mutations in the core promoter (1575–1849, arrow) and precore coding (1814–) regions in viruses from blood samples obtained from HBsAg-positive donors. The blue boxes indicate amino acids from 1610 to 1660 on the left and 1749 to 1780 on the right. The green box indicates amino acids from 1850 to 1901. The blue arrowheads at the top of the nucleotide sequence alignment indicate the positions of the nucleotide mutations. The purple, dark blue, and light blue arrowheads to the left side of the alignment indicate samples with core promoter mutations. The green arrowheads indicate samples with precore mutations. Symbols in the same color as the arrowheads indicate nucleic acid mutations.

The X protein coding region is a core promoter that regulates the expression of the HBe antigen. Core promoter mutations have been reported to accumulate in this area [14, 30–32]. The 1762A>T/1764G>A double mutation, which is the major core promoter mutation, was found in genotypes B and C (Fig. 5), including six of the 10 (60%) genotype C samples (samples 19, 92, 99, 115, 136, and 137), and two of the 15 (13%) genotype B samples (samples 74 and 118). The triple mutation 1653C>T/1762A>T/1764G>A was found in three genotype C samples (samples 19, 99, and 115). One of these samples (sample 19) had a quadruple mutation with 1613G>A. Another triple mutation, 1753T>V/1762A>T/1764G>A, was found in three genotype C samples (samples 82, 136, and 137) and one genotype B sample (sample 74). Two of the genotype C samples (samples 136 and 137) had a quadruple mutation with 1613G>A. A 1613G>A/1653C>T double mutation was found in one genotype B sample (sample 48), accompanied by a 1754T>G mutation. Single 1613G>A, 1753T>V, 1754T>G, 1764G>A, and 1766C>T mutations were found in samples 89, 21, 47, 116, and 130, respectively.

Precore mutations from 1858 to 1896 of the DNA sequence upregulate HBV DNA in serum [15]. All four genotype A HBV DNA samples harbored the 1858T>C mutation. The 1862G>T mutation, which is also critical for regulating hepatitis B e antigen (HBeAg) expression and participates in HBV-related liver injury, was found in one (25%) of the four genotype A and two (13%) of the 15 genotype B samples. The stop codon mutation 1896G>A, which inhibits the production of HBeAg, was found in 10 (67%) of the 15 genotype B and four (27%) of the 15 genotype C samples but was not found in any of the four genotype A samples.

### X protein mutations in HBV DNA-positive blood donor samples

**Fig. 6:**
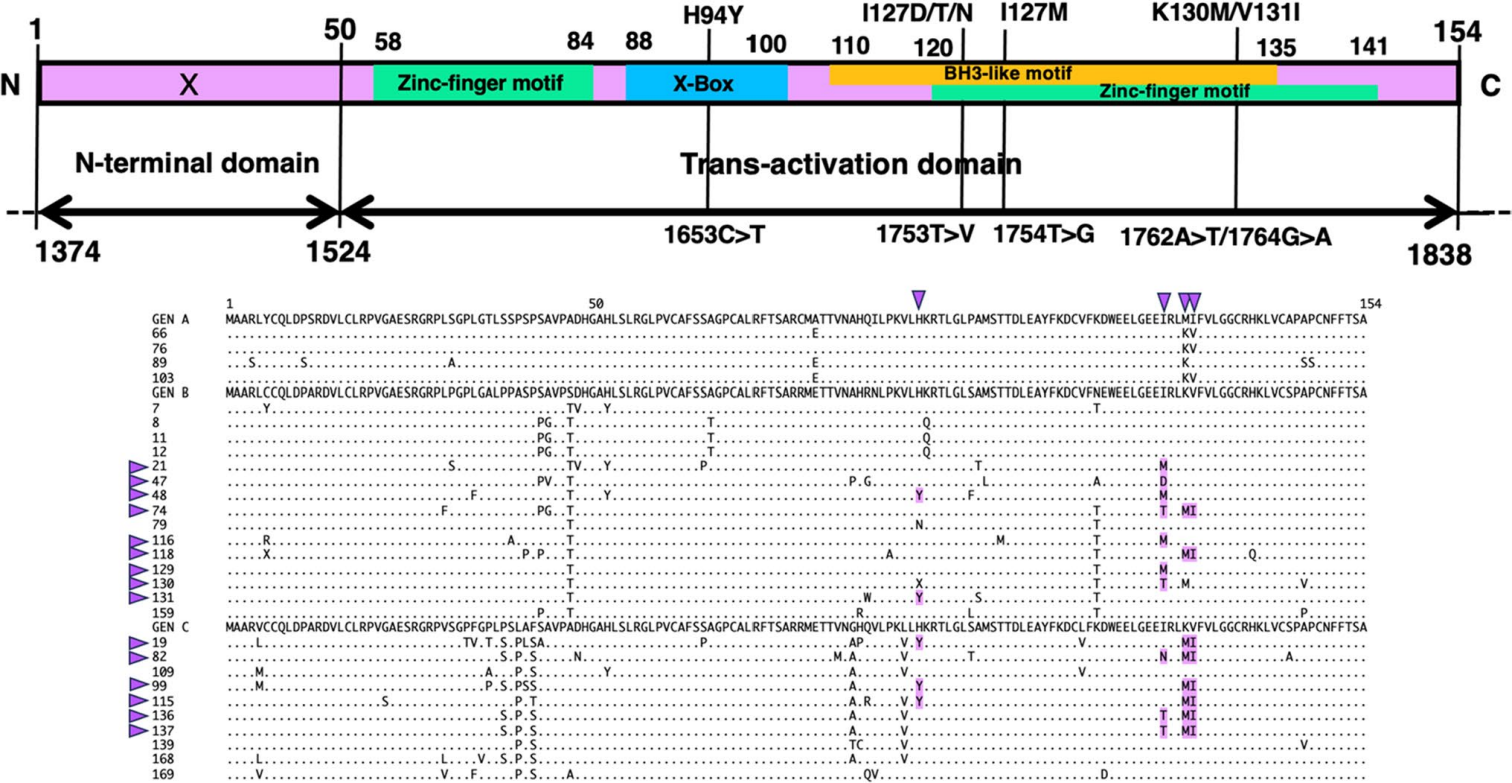
Schematic diagram showing the amino acid sequence of the X protein, comprising the X-box binding domain, BH-3-like motif, and zinc-finger motif indicating the positions of amino acid mutations observed in viruses from blood samples obtained from HBsAg-positive blood donors. The boxes at the top indicate amino acids, and the arrows below indicate nucleotides. The purple symbols indicate amino acid mutations. The purple arrowheads at the top of the amino acid alignment indicate the H94Y, I127D/T/N, I127M, and K130M/V131I mutations. The purple arrowheads to the left of the amino acid alignment indicate samples with amino acid mutations.

The region encoding the HBV-X protein is also a core promoter and is part of the precore region. Therefore, the amino acid sequence of the X protein is mutated in the presence of core promoter or precore mutations. The core promoter mutations 1653C>T, 1753T>V, 1754T>G, and 1762A>T/1764G>A suppress the production of HBeAg and increase the risk of cirrhosis and HCC [15]. We investigated mutations in the amino acid sequence of the X protein in the 29 samples with whole-genome sequences (Fig. 6). An H94Y missense mutation, caused by the core promoter mutation 1653C>T, was found in two (13%) of the 15 genotype B and three (30%) of the 10 genotype C samples. An I127D/T/N missense mutation, caused by 1753 T>V, was observed in three (7%) of the 15 genotype B (7%) and three (30%) of the 10 genotype C samples. An I127M missense mutation caused by 1754T>G was observed in four (27%) of the 15 genotype B samples. The K130M/V131I double mutation due to 1762A>T/1764G>A was found in two (13%) of the 15 genotype B and six (60%) of the 10 genotype C samples.

## Discussion

In this study, HBV DNA was isolated from 58 of the 169 HBsAg-positive samples of blood from Japanese donors, and the region encoding the small envelope protein was sequenced for these 58 samples and analyzed for VEMs. VEMs were identified in six of the 58 samples; these included two K122R/N mutations, two T126A mutations, one M133T mutation, and one F134L mutation; however, no strong VEMs, such as G145R, or multiple VEMs in a single strain were identified. The VEMs observed in this study did not include any missense mutations common to all genotypes. The impact of hepatitis B vaccination on the prevalence of VEMs remains unknown, and there is no definitive demonstration of a causal relationship. In Japan, the universal hepatitis B vaccination was only introduced in 2016, and most people have not yet been vaccinated and do not have antibodies against HBV. Therefore, the results of the VEM analysis in this study provide baseline findings. Future studies must be conducted to investigate whether the prevalence and VEM types change with increasing hepatitis B vaccination coverage, comparing the results obtained with those of this study.

The whole HBV genomes from 29 of the 58 HBV-DNA-positive samples were successfully sequenced. Detailed analysis revealed a greater variety of mutations than expected, including tenofovir DRMs. The finding of tenofovir DRMs in HBV DNA from HBsAg-positive blood samples must be taken seriously [23, 33–37]. Lamivudine, adefovir, entecavir, and tenofovir are used to treat hepatitis B in Japan. However, the absence of personal and medical information for the blood donors who provided the samples prevents the determination of whether the two individuals with DRMs had received hepatitis B treatment in the past. The tenofovir DRMs found in this study could potentially affect the response to treatment.

According to a study conducted by the Japanese Red Cross in 2006 and 2007 [38] that targeted partial sequences of HBV DNA for genotyping, only 3.3% of the 1887 samples from HBsAg-positive blood donors were positive for HBV DNA, of which 19% were genotype A, 16% were genotype B, and 63% were genotype C. PCR targeting the small envelope protein was positive for 34% of the samples in this study. Phylogenetic analysis revealed that 21% were genotype A, 47% were genotype B, and 32% were genotype C. These results suggest that the proportion of genotype B has increased, that of genotype C has decreased, and that of genotype A has remained unchanged among HBsAg-positive blood donors in Japan during the past 15 years.

Most of the mutations found in this study are associated with chronic liver injury. Both of the core promoter mutations identified in this study are important for the regulation of HBeAg expression and are involved in HBV-associated liver injury [15–17, 24, 39–41]. The 1613 mutation decreases the amount of HBeAg and increases that of HBV DNA and is a risk factor for HCC [22, 32, 41]. The double mutation at 1613 and 1653 is associated with a greater increase in the risk of HCC compared with that of the 1613 mutation alone [15, 41]. The double mutation at 1753 and 1899 is a risk factor for cirrhosis. The 1762A>G/1764G>A double mutation and 1753T>V mutation increase the risk of alcoholic liver disease and HCC. These core promoter mutations were particularly common in genotype C [13].

Precore mutations in the region from 1858 to 1896 increase the HBV DNA levels in serum. Three cases of the 1862G>T mutation, which is a risk factor for FH and acute liver failure (ALF), were identified [17, 18]. Nonsense mutations in 1896 result in the loss of HBe antigen production. The reduction in HBe antigen levels induces hepatocyte apoptosis and regeneration, leading to liver injury [15, 19, 42, 43]. Concurrent double mutation at 1762 and 1764 in the core promoter region and the 1896G>A stop codon mutation in the precore region was identified in two cases in genotype C and in one case of genotype B infection. This combination of mutations is associated with an increased risk of HBV-associated liver injury [15, 18, 44]. Concurrent mutation 1753T>V (C, G, A) in the core promoter and the stop codon mutation 1896G>A in the precore region also increases the risk of FH and ALF [17, 20]. This mutation was found in two cases of genotype B infection. None of the four genotype A strains had core promoter mutations that could cause such HBV-associated liver damage, but all had the 1858T>C mutation. The 1858T>C mutation prevents the 1896G>A mutation [42]. No mutations at the 1986 position were found in this study.

The HBV receptor recognition domain is located within amino acids 2–48, counting from the N-terminus of the large envelope protein preS1 [45–48], and amino acids 9–18 in particular are essential for the HBV receptor function [26]. Alignment of the amino acid sequence of preS1 revealed that the virus in sample 89 had a deletion mutation in amino acids 1–11, which includes the region that is critical for the binding of HBV to hepatocytes.

The HBsAg-positive blood samples in this study were provided by the Japanese Red Cross Society. For ethical reasons, we could not obtain age, sex, or medical information for the blood donors, except for the blood type. Therefore, a limitation of this study is that we could not determine whether the HBV-infected blood donors with HBV mutations could be at risk of HBV-associated chronic liver injury or whether those with tenofovir-resistant HBV mutations had a history of treatment for acute HBV infection. Additionally, assessing the duration elapsed since the initial infection was not possible, as blood donors may not have been aware that they were HBV carriers while cooperating with blood donations. The 1896G>A mutation occurs after a longer period of infection, and antibodies against HBe are produced earlier in genotype B infections than in genotype C [19].

An advantage of using donated blood samples for this study was that phylogenetic analysis and sequence comparisons could be performed on asymptomatic carriers who had not been diagnosed with HBV infection or treated for HBV. However, not all HBV-DNA-positive blood donors are necessarily untreated carriers because some may be unaware that a history of HBV treatment makes them ineligible for blood donation, some may be unaware of the disease, and some may use blood donations as a substitute for HBV testing. In addition, because the specimens for this study were obtained from the Japanese Red Cross Kanto-Koshinetsu Block Blood Center, which collects blood donations from Tokyo and two surrounding prefectures, the results may not accurately reflect the current situation in the whole of Japan. These limitations should be considered when interpreting the results.

In conclusion, HBV DNA was isolated from samples collected from Japanese HBsAg-positive blood donors and subjected to whole-genome sequencing to identify VEMs, tenofovir DRMs, and mutations associated with chronic liver injury. The finding of mutations, including DRMs, in multiple samples from blood donors was unexpected. This study provides a baseline for continued surveillance of mutation trends in HBsAg-positive blood donors in Japan. We suggest that a clinical study to identify VEMs and DRMs in HBsAg-positive individuals needs to be conducted.

### Supplementary Information

Below is the link to the electronic supplementary material.

Supplementary file1 (PPTX 134 KB)

## Data Availability

All HBV DNA sequences identified in this study are registered in the DNA Data Bank of Japan (DDBJ), and sequencing data can be accessed by accession number.
