# Supplementary material for: Characterization of mutations in hepatitis B virus DNA isolated from Japanese HBsAg-positive blood donors in 2021 and 2022
Source: Arch Virol. 2024 Apr 18;169(5):103. doi: 10.1007/s00705-024-06016-4 (PMC11023964; doi:10.1007/s00705-024-06016-4)
Supplement: Supplementary file 1 — Supplementary file1 (PPTX 134 KB) [file 705_2024_6016_MOESM1_ESM.pptx]

## Slide 1
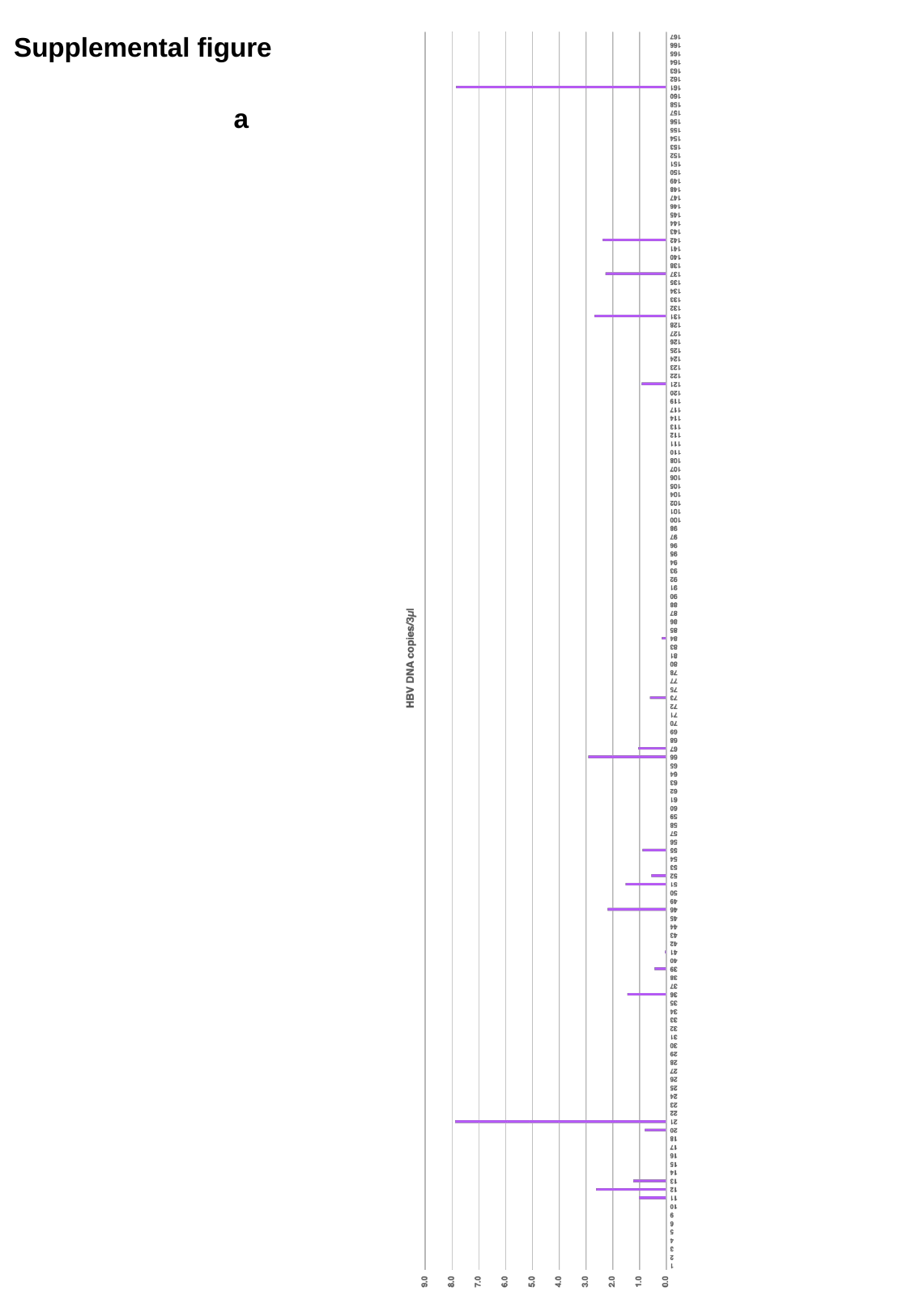

Supplemental figure
a

## Slide 2
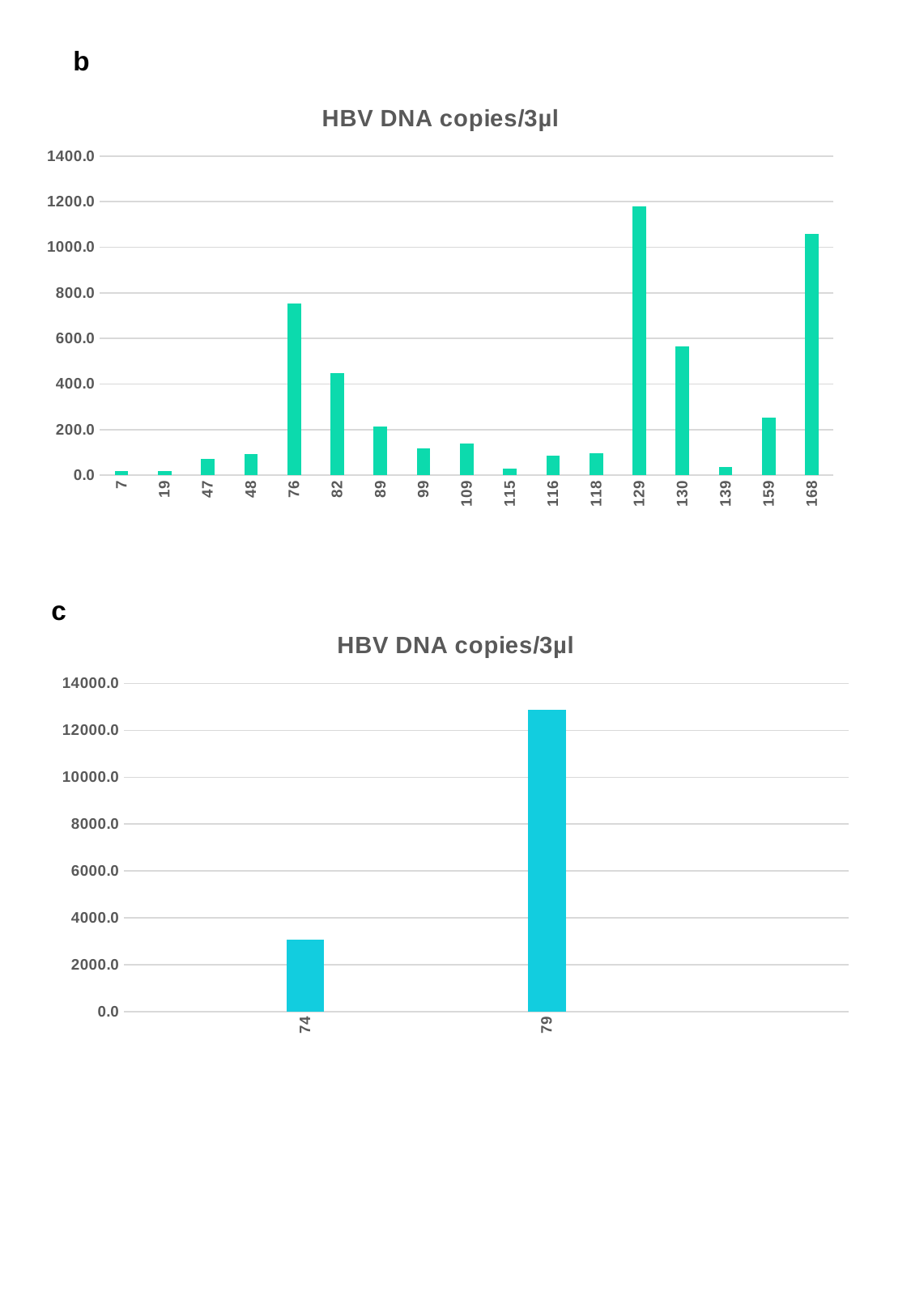

b
### Chart: HBV DNA copies/3µl
| Category | |
|---|---|
| 7 | 17.8333264872046 |
| 19 | 18.265528310606 |
| 47 | 72.9806117647391 |
| 48 | 92.5098138218934 |
| 76 | 754.930471482574 |
| 82 | 449.548667789036 |
| 89 | 212.861232547919 |
| 99 | 117.503643696254 |
| 109 | 140.857119104951 |
| 115 | 30.7682571661856 |
| 116 | 86.3795796081557 |
| 118 | 94.704876503595 |
| 129 | 1177.81638022969 |
| 130 | 566.672630734119 |
| 139 | 36.5739398181687 |
| 159 | 254.132392379199 |
| 168 | 1057.44554357484 |c
### Chart: HBV DNA copies/3µl
| Category | |
|---|---|
| | None |
| 74 | 3065.95010213031 |
| | None |
| 79 | 12864.8633143797 |
| | None |
| | None |

## Slide 3
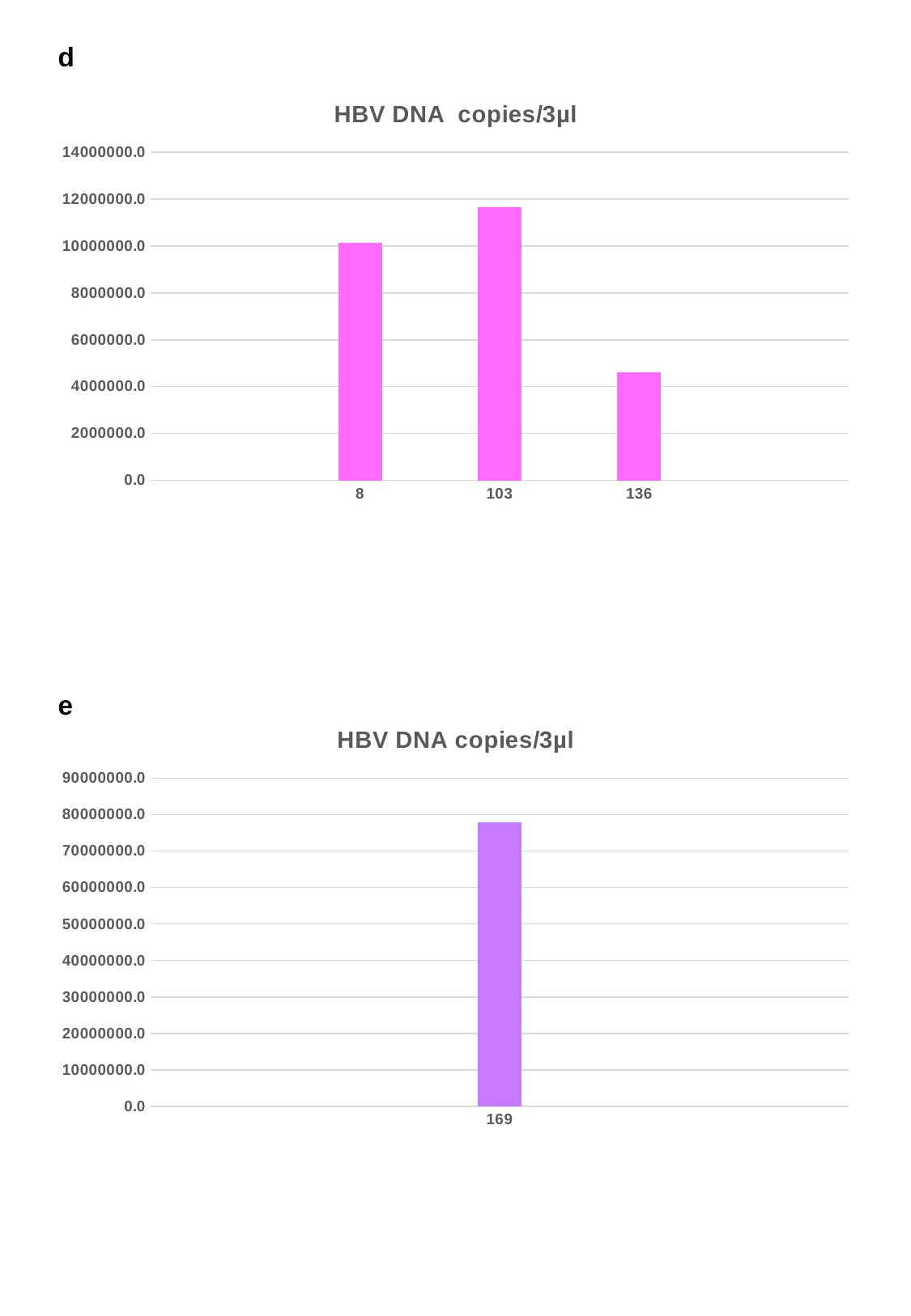

d
### Chart: HBV DNA copies/3µl
| Category | |
|---|---|
| | None |
| 8 | 10128493.5144984 |
| 103 | 11642793.0524712 |
| 136 | 4613075.56537688 |
| | None |e
### Chart: HBV DNA copies/3µl
| Category | |
|---|---|
| | None |
| | None |
| 169 | 77989393.0654319 |
| | None |
| | None |
